# Bioinformatic challenges for pharmacogenomic study: tools for genomic data analysis

**DOI:** 10.3389/fphar.2025.1548991

**Published:** 2025-04-11

**Authors:** Mariamena Arbitrio, Marianna Milano, Maria Lucibello, Emanuela Altomare, Nicoletta Staropoli, Pierfrancesco Tassone, Pierosandro Tagliaferri, Mario Cannataro, Giuseppe Agapito

**Affiliations:** ^1^ Institute for Biomedical Research and Innovation, National Research Council, Catanzaro, Italy; ^2^ Department of Experimental and Clinical Medicine, University Magna Græcia, Catanzaro, Italy; ^3^ Medical Oncology Unit, R. Dulbecco (Mater Domini Facility), Teaching Hospital, Magna Græcia University and Cancer Center, Campus Salvatore Venuta, Catanzaro, Italy; ^4^ Department of Health Science, University Magna Græcia, Catanzaro, Italy; ^5^ Department of Medical and Surgical Sciences, University Magna Græcia, Catanzaro, Italy; ^6^ Department of Law, Economics and Social Sciences, University Magna Græcia, Catanzaro, Italy

**Keywords:** pharmacogenomics, genomic data analysis, bioinformatics, biological pathways, network analysis, pathway enrichment analysis

## Abstract

The sequencing of the human genome in 2003 marked a transformative shift from a one-size-fits-all approach to personalized medicine, emphasizing patient-specific molecular and physiological characteristics. Advances in sequencing technologies, from Sanger methods to Next-Generation Sequencing (NGS), have generated vast genomic datasets, enabling the development of tailored therapeutic strategies. Pharmacogenomics (PGx) has played a pivotal role in elucidating how the genetic make-up influences inter-individual variability in drug efficacy and toxicity discovering predictive and prognostic biomarkers. However, challenges persist in interpreting polymorphic variants and translating findings into clinical practice. Multi-omics data integration and bioinformatics tools are essential for addressing these complexities, uncovering novel molecular insights, and advancing precision medicine. In this review, starting from our experience in PGx studies performed by DMET microarray platform, we propose a guideline combining machine learning, statistical, and network-based approaches to simplify and better understand complex DMET PGx data analysis which can be adapted for broader PGx applications, fostering accessibility to high-performance bioinformatics, also for non-specialists. Moreover, we describe an example of how bioinformatic tools can be used for a comprehensive integrative analysis which could allow the translation of genetic insights into personalized therapeutic strategies.

## 1 Introduction

The sequencing of the human genome in 2003 revolutionized medicine, shifting from a one-size-fits-all approach for disease treatment toward personalized medicine based on patient-specific molecular and physiological characteristics, as well as susceptibility to specific diseases or responsiveness to the same treatments. At the same time, advancements in sequencing technologies—from the Sanger method and high-throughput approaches to deep sequencing by Next-Generation Sequencing (NGS)—have generated a vast amount of complex genomic data, significantly improving personalized medicine (Scionti et al., 2022). Consequently, new concepts in “omic sciences” have been introduced and developed, including genomics, transcriptomics, proteomics, and metabolomics, enhancing our understanding of complex molecular interactions at various biological system levels (Dai and Shen, 2022). In this context, the study of inter-individual differences in drug efficacy and toxicity in response to the same treatment has impacted several therapeutic areas, including neurology, psychiatry, cardiology, and analgesia, with significant implications for cancer treatment. These innovations have facilitated a deeper understanding of cancer heterogeneity and complexity at cellular and molecular levels, enabling the identification of druggable gene aberrations and predictive, diagnostic, and prognostic biomarkers that have improved patient outcomes. Pharmacogenomics (PGx) helps elucidate how inherited genetic backgrounds influence the unpredictable inter-individual variability in drug response within the framework of precision medicine (Arbitrio et al., 2023; Arbitrio et al., 2021; Arbitrio et al., 2018; Arbitrio et al., 2022). PGx studies have contributed to identifying genetic variants linked to patient variability in drug response, particularly in pharmacokinetics (PK) and pharmacodynamics (PD), leading to the discovery of “agnostic” predictive and prognostic biomarkers. However, the clinical-grade development of biomarkers requires a rigorous validation process under stringent regulatory guidelines (Arbitrio et al., 2021). Despite technological advancements that have improved patient sequencing knowledge and data availability, analyzing and interpreting polymorphic variants and translating them into clinical practice remains a challenge. It requires interdisciplinary efforts for the large-scale implementation of preemptive precision medicine programs (Guzzi et al., 2016). The integration of large multi-omics datasets has the potential to uncover complex molecular interactions and pathways, paving the way for significant advancements in personalized medicine, drug discovery, and disease mechanism understanding. Achieving this goal necessitates using diverse bioinformatics tools to build software pipelines tailored to specific data types. These approaches enable researchers to address challenges related to complexity, heterogeneity, integration, and harmonization of high-throughput omics data. Notably, no single analytical methodology is sufficient for analyzing all available datasets, as a one-size-fits-all approach rarely meets the intricate demands of multi-omics data analysis (Pastrello et al., 2013).

Bioinformatics tools play a crucial role in managing, analyzing, and interpreting large-scale and complex genomic datasets. These tools facilitate the integration of diverse omics layers, allowing researchers to bridge knowledge gaps and derive meaningful biological insights. In PGx, analyzing genetic variants that influence drug metabolism requires robust computational methods. One widely used platform for this purpose was the DMET (Drug Metabolism Enzymes and Transporters) Plus microarray platform, developed by Thermo Fisher Scientific Inc (Waltham, MA, United States), which studied 1,936 markers recognized by the FDA for their role in drug metabolism (Staropoli et al., 2022; Giannitrapani et al., 2024; Staropoli et al., 2024). While the DMET console software provides raw data, additional bioinformatics techniques are required for data interpretation and clinically relevant insights.

To address this challenge, we have developed a comprehensive guideline for analyzing genomic data, drawing from our experience with DMET and SNP datasets. This process integrates multiple analytical approaches, including statistical techniques, machine learning algorithms, network analysis, and pathway enrichment tools. By combining these methodologies, our approach enables systematic, high-performance analysis, annotation, and integration of PGx data, ensuring accessibility even for non-specialists in bioinformatics. This structured workflow enhances the reliability of PGx studies, promoting the translation of genetic insights into actionable clinical decisions.

In this review, we emphasize the importance of PGx in clinical practice and the necessity of bioinformatics approaches for analyzing large-scale PGx datasets. Furthermore, to promote the widespread adoption of bioinformatics in clinical settings, it is essential to extract actionable knowledge from PGx repositories. To this end, we present a selection of available PGx databases curated and evaluated by experts. Additionally, we introduce existing network-based bioinformatics tools and present a carefully curated selection of machine learning, statistical frameworks, and pathway enrichment analysis approaches to analyze complex DMET PGx data. In addition, a general protocol for PGx dataset analysis is provided, demonstrating how bioinformatics can be leveraged to enhance precision medicine. Moreover, we discuss how the integration between bioinformatics and PGx can contribute to precision medicine.

## 2 Implementation of PGx knowledge into clinical practice

Polymorphic variants in drug-metabolizing enzymes and transporters (ADME genes), such as phase 1 CYP450 enzymes (e.g., CYP3A4, CYP2C8, CYP2D6), phase 2 enzymes [e.g., Uridine 5′-diphospho-glucuronosyltransferases (UGTs), aldehyde oxidase (AO), N-acetyltransferase (NAT), flavin monooxygenase (FMO)] and transporters [e.g., ATP binding cassette (ABC), Breast Cancer Resistance Protein (BCRP), organic anion transporting polypeptides (OATPs), Solute carrier transporters (SLC)] could contribute substantially to PK/PD variability. PGX studies highlight the role of polymorphisms in ADME genes in the modulation of the exposure, efficacy, and safety of a drug, suggesting dose adjustments and recommendations for specific populations. Regulatory agencies like the FDA, EMA have recognized and inserted in the drug labeling PGx information related to strong genotype-phenotype evidence, to follow for therapeutic management (Arbitrio et al., 2021). The most common ADME germline variants, inducible and/or polymorphic, involved in impaired or enhanced drug biotransformation are single nucleotide polymorphisms (SNPs), copy number variations (CNVs), insertions, deletions and a variable number of tandem repeats (Ismail and Essawi, 2012). SNPs are common inherited variations (>1%) among people located in haplotype blocks separated by areas of hyper-recombination sites (Hot Spot) and in strong Linkage Disequilibrium (LD) with specific polymorphic variants in other ADME genes, which can be used as markers of a particular haplotype (Tag-SNPs) (Huang et al., 2006; Arbitrio et al., 2018). Well-known examples of relevant SNPs in metabolic enzymes and transporters associated with recommendations for dose modification are CYP2C $19^{*}$ 17, associated with bleeding during clopidogrel therapy, and VKORC1 variants associated with warfarin resistance; DPYD variants (DPYD*2A (c.1905+1G > A), c.2846A > T: loss-of-function), correlated to toxicity risk of 5-fluorouracil or capecitabine; TPMT variants (TPMT*2, *3A, *3C: reduced activity) linked to thiopurine-induced myelosuppression, UGT1A*28, associated to gastrointestinal irinotecan toxicity; SLCO1B $1^{*}$ 5 variant correlated to high risk for simvastatin toxicity; ABCG2 (BCRP) variants (c.421C > A (Q141K): reduced activity) linked to chemoresistance or HLA-B*5,701 associated to the risk of abacavir hypersensitivity.

Until now, in clinical practice, according to regulatory guidelines, the PGX test for the research of genetic polymorphisms is highly suggested before starting therapy with these drugs. All information and data/recommendations on allelic variants of ADME genes are rigorously curated and evaluated by experts and available in comprehensive databases and resources, updated periodically, and freely available, such as the pharmacogenomics knowledge implementation (PharmGKB) (Thorn et al., 2005), the Clinical Pharmacogenetics Implementation Consortium (CPIC) (Relling et al., 2020) in United States and the Dutch Pharmacogenetics Working Group (Bank et al., 2018) (Europe, E and Netherlands, NL), the dbSNP (Sherry et al., 2001) (Database of Single Nucleotide Polymorphisms), the PharmVar (Gaedigk et al., 2021) (Pharmacogene Variation Consortium)and DrugBank (Wishart et al., 2018). These databases provide curated information on gene-drug interactions, allelic variants, and clinical guidelines in the perspective of precision medicine with specific characteristics and differences, as reported in Table 1.

**TABLE 1:** Databases features.

Database	Focus	Features	Strengths	Limitations
PharmGKB	PGx knowledge base	Drug-gene pairs, guidelines, pathway maps	Comprehensive, links to CPIC	Limited to well-studied variants
CPIC (United States)	Clinical implementation	Dosing guidelines based on variants	Actionable recommendations	Limited gene-drug pairs
DPWG (E, NL)	PGx guideline for clinical use	Dosing guidelines based on variants	Actionable recommendations	Dutch healthcare system, limited scope in gene-drug pairs
dbSNP	Genetic variants	SNP details, phenotype links	Comprehensive variants database	No drug-specific data
DrugBank	Drug data	Drug actions, targets, PGx interactions	Broad scope, detailed drug profiles	Limited focus on PGx
PharmVar	Pharmacogene variants	Star allele nomenclature	Standardized allele information	Limited number of genes

The table points out the focus, the features, the strengths, and the limitations of each listed database.

Table 2 summarizes for each mentioned database the number of Clinical Annotations, VIPs, and Variants provided.

**TABLE 2:** Database statistics.

Database	CA	VIPs	VA	log2 (CA)	log2 (VIPs)	log2 (VA)
PharmGKB	5,180.00	34.00	28,298.00	12.34	5.09	14.79
CPIC	0.00	0.00	0.00	0	0	0
DPWG	0.00	0.00	0.00	0	0	0
dbSNP	2,076,313.00	0.00	2,618.00	20.99	0	11.35
DrugBank	2,812.00	0.00	5,467.00	11.46	0	12.42
PharmVar	0.00	0.00	15.00	0	0	3.91

Database statistics for Clinical Annotations, VIPs, and Variants. PharmGKB has the highest variant annotations (28,298). dbSNP has the largest number of clinical annotations (over 2 million). DrugBank contains 2,812 clinical annotations and 5,467 variant annotations. CPIC and DPWG do not contain clinical or variant annotations because they provide clinical and therapeutic recommendations. In the table, CA means Clinical Annotations, VIP is short for mean Very Important Pharmacogenes and VA indicates Variant Annotations.

**FIGURE 1:**
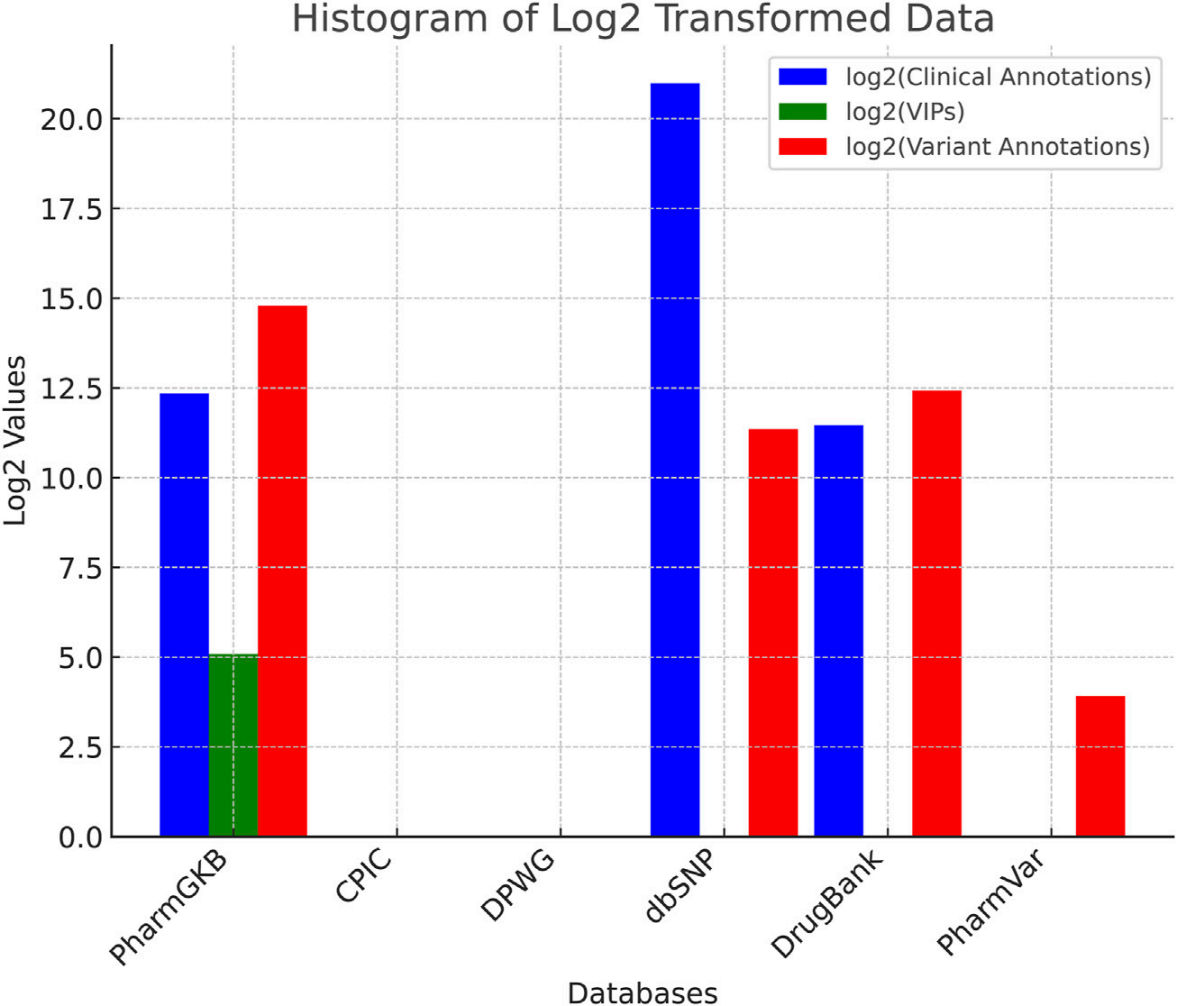
Normalized comparison of the mentioned databases, highlighting their relative strengths regarding the number of Clinical Annotations, Very Important Pharmacogenes (VIPs), and Variant Annotations for each database.

To effectively visualize the data, we apply a logarithm base 2 $\left(log_{2}\right)$ transformation to address the significant differences in magnitude among various databases. The dataset ranges from 15 variant annotations (PharmVar) to over 2 million clinical annotations (dbSNP), which leads to high skewness. Without transformation, smaller values would be nearly imperceptible against larger ones. The $log_{2}$ transformation compresses large values while maintaining relative differences, enhancing comparison across datasets. By using $log_{2}$ , we improve the readability and interpretability of the data, making variations across all categories clear in a single graphical representation. Figure 1 visualizes the number of Clinical Annotations, VIPs, and Variant Annotations for each database.

The PharmGKB (http://www.pharmgkb.org/) is a comprehensive database on gene-drug interactions, allelic variants, annotations on critical pharmacogenetic (VIP), drug pathways, and their relationship with drug response as well as ADRs to help researchers understand how polymorphic variants can affect drug PK or PD. All data and each annotation are mainly derived from PGx studies integrating information from the CPIC to provide drug-dosing guidelines according to personal genotype.

The CPIC (https://cpicpgx.org/) aims to provide actionable recommendations for drug dosing based on genetic variants, especially on pharmacogenes, with strong evidence, giving practice guidelines to allow the introduction of PGx research into clinical practice. All information is collected from biological research to clinical studies and incorporated into the guidelines, linking genotypes with phenotypes to help clinicians understand how a genetic test can optimize drug therapy.

The DPWG (www.knmp.nl) is a leading organization that gives guidelines to integrate pharmacogenomics tests into clinical practice with actionable recommendations in PGx practices in Europe but with a strong focus on the Netherlands. Its primary focus is to optimize drug therapy, suggesting whether and when genetic testing should be conducted. Compared to CPIC, DPWG may lack depth in specific less-studied gene-drug pairs. By collaborating with other international consortia, the DPWG contributes to harmonizing guidelines.

The DrugBank (https://go.drugbank.com) is a comprehensive resource that provides information on mechanisms, pharmacology, PGx, drug-food and drug-drug interactions and targets of selected FDA-approved drugs. This database helps understand drug mechanisms and targets, facilitating silico drug design. In comparison, PharmGKB is specialized in PGx-specific annotations.

The dbSNP database (http://www.ncbi.nlm.nih.gov/SNP) is a dense, central, public repository catalog of genetic variants, including SNPs, constructed by the National Center for Biotechnology Information (NCBI) in collaboration with the National Human Genome Research Institute (NHGRI). However, the database does not provide specific drug-gene interactions data.

The PharmVar Consortium (https://www.pharmavar.org) is the new data repository for PGx gene nomenclature to catalog a limited number of allelic variants of ADME genes which provide a universal star (*) nomenclature and serve as a centralized “Next-Generation” Pharmacogene Variation data repository for genes involved in drug metabolism as well as for genes contributing to drug transport and response. PharmVar provides several graphical display functions and custom tables showing genes or gene regions of interest for SNPs that are important for interpreting genotyping results.

Although there is objective evidence that ADME genotyping significantly contributes to the appropriate and safe prescription of drugs, the preemptive introduction of PGx in clinical daily practice is still very low. Genetic testing is routinely used only for drugs with labeling recommendations. Although clinicians might be supported by PGX guidelines developed after multidisciplinary efforts, several other barriers (ethical, legal, social) must be overcome. All efforts that can be made to demonstrate the usefulness and cost-effectiveness of the PGx test in routine practice could represent essential advantages for human health. Moreover, bioinformatic integration by algorithms of both clinical and genetic factors in drug prescribing could significantly help PGx implementation.

## 3 Statistical and machine learning tools for PGx

The increasing availability of genomic, clinical, and pharmacological data has driven the integration of machine learning (ML) techniques to uncover complex patterns and improve predictive models for drug efficacy and adverse reactions. ML tools play a crucialrole in PGx by identifying genetic biomarkers, optimizing drug selection, and predicting patient-specific treatment outcomes. This section explores key machine learning methodologies applied in PGx, ranging from traditional statistical models to advanced deep learning frameworks, highlighting their contributions, challenges, and future directions in precision medicine.

### 3.1 Statistical framework to analyze DMET data

DMET-Analyzer (Guzzi et al., 2012) is an advanced tool designed to streamline the analysis of drug metabolism and transport data obtained from the DMET microarray assay. DMET-Analyzer can be freely downloaded at https://sourceforge.net/projects/dmetanalyzer/files/. By automating the association analysis between genetic variations (SNPs) and clinical conditions, particularly drug responses, it simplifies complex workflows, minimizes errors, and delivers fast, accurate results. A key strength of the DMET-Analyzer is its ability to efficiently process large datasets while automatically annotating significant SNPs and linking them to external databases like dbSNP and PharmaGKB. This feature enhances biological insights and ensures access to up-to-date scientific knowledge. Additionally, its user-friendly interface allows even non-specialists to perform statistical tests, such as Fisher’s exact test and Hardy-Weinberg equilibrium calculations, with ease. The tool identifies genetic markers associated with drug efficacy and adverse reactions, supports SNP visualization through heatmaps, and employs robust statistical corrections like Bonferroni and False Discovery Rate adjustments.

CoreSNP (Guzzi et al., 2013) is the parallel version of DMET-Analyzer. It focuses on massively statistical analysis of SNP data, introducing advanced features such as parallel execution of preprocessing and statistical computations, enabling faster handling of large datasets. CoreSNP automates the entire workflow, from managing microarray input files to conducting statistical association tests like the Fisher test, making it more accessible to biologists while retaining high scalability and efficiency.

Cloud4SNP (Agapito et al., 2013) is a cloud-DMET-Analyzer version, a bioinformatics tool designed for parallel processing and statistical analysis of Single Nucleotide Polymorphisms (SNPs) data, with a focus on PGx. It efficiently manages large-scale PGx datasets, addressing computational challenges in analyzing genetic variations and drug responses. As a Software-as-a-Service (SaaS) solution, Cloud4SNP eliminates the need for local installations, offering a web-based interface that broadens access to high-performance computing resources. This democratizes PGx research, benefiting smaller research groups, while also introducing challenges related to sensitive data security in healthcare organizations (Agapito and Cannataro, 2023).

OSAnalyzer (Agapito et al., 2016) is a bioinformatics tool designed to analyze single polymorphisms and their correlation with clinical outcomes, particularly in cancer patient data. It is optimized for processing large omics datasets that assess gene variants involved in drug metabolism. OSAnalyzer features a powerful visualization engine for Kaplan-Meier curves and statistical metrics such as medians, hazard ratios, and log-rank tests to evaluate overall survival (OS) outcomes. It supports multiple data formats, including Excel and CSV, facilitating seamless data integration. The tool automates survival analysis by computing and visualizing survival curves for genetic variants, presenting results in an intuitive manner. By integrating clinical annotations, OSAnalyzer establishes connections between genetic variants and patient outcomes, including OS and Progression-Free Survival (PFS). Users can prioritize findings by ranking probes based on p-values, enabling the identification of clinically significant variants. Additionally, OSAnalyzer enhances interpretation by linking genomic data with external resources such as dbSNP and PharmaGKB. The tool streamlines data preprocessing, allowing users to rapidly derive actionable insights while ensuring accuracy and reproducibility. OSAnalyzer is available for download at: https://sites.google.com/site/overallsurvivalanalyzer/home.

Researchers can utilize the specific framework for her/his needs to identify critical genetic variants affecting drug efficacy, while clinicians can leverage this information to customize treatments based on patients’ genetic profiles, reducing side effects and enhancing therapeutic effectiveness.

### 3.2 Data mining framework to analyze DMET data

DMET-Miner (Agapito et al., 2015) is a software platform specifically designed to analyze PGx data generated by the Affymetrix DMET platform. DMET-Miner is available for download at https://sites.google.com/site/dmetminer/related-work. Its core functionality lies in the efficient discovery of association rules that highlight multifactorial links among single nucleotide polymorphisms (SNPs) and their correlation with clinical conditions. Unlike its predecessor, DMET-Analyzer, which was limited to identifying individual genetic variants, DMET-Miner employs advanced data mining methodologies to uncover complex relationships among multiple SNPs.

DMET-Miner investigates DMET datasets using frequent itemset mining and optimized association rule extraction. By implementing an enhanced FP-Growth algorithm, DMET-Miner excels in handling high-dimensional datasets, reducing execution time and memory consumption while maintaining the biological relevance of its findings. It also seamlessly integrates with external genomic databases such as dbSNP and PharmaGKB, offering enriched annotations and broader contextual understanding of the extracted rules. DMET-Miner’s user-graphic interface and advanced algorithms streamline data interpretation, enabling users to quickly identify clinically significant genetic markers and their potential impact on drug efficacy or adverse reactions. DMET-Miner can be used in various real-world scenarios, such as drug development, to identify genetic factors and multifactorial relationships that contribute to variability in drug responses. This capability enables the design of safer and more effective pharmaceuticals.

PARES (Parallel Association Rules Extractor from SNPs) (Agapito et al., 2019) and BalancedPARES (BPARES) (Agapito et al., 2021) are an optimized parallel implementation of DMET-Miner, designed to efficiently process genomic data. The PARES and BPARES applications can be downloaded from the following links https://sites.google.com/site/pareswebsite/. In addition, CloudDmet-Miner, an adaptation of DMET-Miner for Amazon Server less Lambda, facilitates efficient SNP dataset analysis on AWS (Crespo-Cepeda et al., 2019). It utilizes serverless computing for dynamic resource scaling and parallel processing, leading to reduced execution times.

In clinical settings, it aids in customizing treatments by integrating genetic profiles to minimize adverse drug reactions and optimize therapeutic outcomes. For academic researchers, DMET-Miner is a powerful tool for exploring single and multifactorial genetic influences on pharmacokinetics and pharmacodynamics.

Table 3 summarizing the main characteristics between DMET-Analyzer, DMET-Miner, and OSAnalyzer in terms of their features, usage, and capabilities.

**TABLE 3:** Statistical and ML tools features.

Tool	Function	Data input	Output	Key features	Use case
DMET-Analyzer	Statistical Analysis of DMET data for pharmacogenomics research	DMET array data	statistical relevant probes	Focuses on genotyping data from DMET chips	Identifying single genetic variations affecting drug metabolism and efficacy
DMET-Miner	Facilitates mining and interpretation of pharmacogenomic data from DMET arrays	DMET array data	Multiple association data	Emphasis on association rule mining	Researching correlations between multiple genetic variations and drug responses
OSAnalyzer	Performs OS analysis using DMET data annotated with clinical information	DMET annotated data set	OS curves sorted by p-value significance	Enables OS analysis of DMET data annotated with survival data	Exploration of pharmacogenomic impacts on patient outcomes

Main characteristics of DMET-Analyzer, DMET-Miner, and OSAnalyzer.

## 4 Network analysis in pharmacogenomics

Network analysis is a branch of network science focused on the study of complex networks. To investigate intricate relationships, network analysis employs theories and methods from various research domains (Milano and Cannataro, 2023). Networks and network analysis methods are foundational in computational biology and bioinformatics, where they are increasingly applied to study biological and clinical data in an integrative manner.

Specifically, network analysis comprises a suite of techniques with an unified methodological perspective, enabling the depiction of relationships among entities and the analysis of emergent structures from the recurrence of these relationships. The core assumption is that analyzing these interconnections yields more comprehensive explanations of diverse phenomena. One prominent method in network analysis is community detection (Fortunato and Hric, 2016).

Community detection is a major area of research across various complex systems, including biology, sociology, medicine, and transportation (Lancichinetti and Fortunato, 2012; Gligorijević et al., 2016). Community structures, defined as groups of nodes that are more densely connected than the rest of the network, are particularly significant for understanding the functionality and organization of complex systems modeled as networks (Fortunato and Hric, 2016). Communities are expected to play critical roles in the relationship between structure and function.

For example, in biological networks such as Protein-Protein Interaction (PPI) networks, communities often represent proteins involved in similar functions. In neuroscience, communities detected in brain networks correspond to regions of interest (ROIs) active during specific tasks. In social networks, communities represent groups such as friends or colleagues. On the World Wide Web, communities are often groups of web pages sharing the same topic (Wang et al., 2018). As a result, discovering communities in such systems has become a compelling approach to understanding how network structures relate to system behaviors.

In recent years, network analysis has gained prominence in PGx (Zhou and Lauschke, 2020; Shiota et al., 2008). By providing a powerful framework for modeling data, network analysis enables researchers to analyze and interpret complex interactions between genes, proteins, and drugs. This facilitates the uncovering of underlying biological mechanisms, identification of potential drug targets and biomarkers, promotion of drug repurposing, and support for personalized medicine approaches. Applications of network analysis in PGx encompass several transformative areas, offering a robust framework for understanding complex biological interactions. One key application is the identification of drug targets. By constructing biological networks that integrate diverse data sources, such as protein-protein interactions, gene expression profiles, and pathway information, network analysis allows for the identification of crucial genes or proteins within disease pathways or drug response mechanisms. Topological analyses and the identification of key nodes or modules within these networks provide valuable insights that can guide the development of targeted therapies (Kushwaha and Shakya, 2010; Hasan et al., 2012; Iorio et al., 2010).

Another important application is biomarker discovery. By integrating genomic and clinical data, researchers can create networks that capture relationships between genetic variations, clinical phenotypes, and drug responses. These networks can highlight specific modules or subnetworks strongly associated with particular drug responses, facilitating the identification of biomarkers that enable personalized medicine approaches (Giacomini et al., 2007).

Network analysis also plays a significant role in pathway analysis by revealing interconnected biological pathways and processes affected by genetic variations or drug treatments. Mapping genetic variants onto biological networks enables researchers to identify pathways enriched with these variants, providing deeper insights into molecular mechanisms and highlighting potential targets for therapeutic intervention (Milano et al., 2022a).

Moreover, network analysis supports drug repurposing efforts and the identification of off-target effects. By examining the interactions between drugs, genes, and diseases within a network context, researchers can uncover potential secondary uses for existing drugs or identify unintended effects that may arise during treatment (Lotfi Shahreza et al., 2018).

Finally, network analysis facilitates advancements in personalized medicine by incorporating patient-specific genetic and clinical data into networks. This integrative approach enables the development of tailored treatment strategies, addressing individual patient needs and improving therapeutic outcomes. The increasing complexity of real-world systems has prompted the development of multilayer networks as an extension of traditional graph theory. Classical network approaches often fail to comprehensively capture the intricacies of many systems, necessitating more advanced frameworks (Boccaletti et al., 2014; Milano et al., 2022b).

Multilayer networks provide a richer and more realistic representation of systems with multiple types of relationships. This approach enables the analysis and understanding of dynamics and behaviors of interconnected entities in a more nuanced way (Kinsley et al., 2020).

Multilayer network analysis allows the study of properties and phenomena not easily captured by traditional methods. For example, it enables examination of interdependencies, correlations, and patterns across different layers. Insights can include how layers influence each other, system resilience, the spread of information or diseases, and the identification of key nodes or communities in multilayered systems (Milano et al., 2023).

### 4.1 Network alignment algorithms for pharmacogenomics

Network alignment (NA) is a computational technique widely used for comparative analysis of PPI networks between species, in order to predict evolutionary conserved components or sub-structures in a system data level. Network alignment is a common problem that requires to search a node mapping that best fits one network into another network. Here we reported two aligment algorithm that can be applied to pharmacogenomics data.

### 4.2 L-HetNEtAligner

L-HetNEtAligner Milano et al. (2020) is an advanced algorithm for aligning multilayer heterogeneous networks, addressing the complexity of such networks by incorporating both topological and semantic information from multiple layers. The algorithm combines structural alignment, which evaluates the similarity of node neighborhoods and interconnections across layers, with semantic alignment, leveraging node attributes such as functional annotations, biological roles, or other relevant metadata. This dual-focus approach ensures that the alignment is both structurally consistent and contextually meaningful.

The process begins by computing initial similarity scores between nodes based on their topological properties and semantic attributes. These scores are integrated into an objective function designed to balance the contributions of topology and semantics. L-HetNEtAligner then employs an iterative optimization procedure to refine the alignments, updating the similarity scores to account for newly discovered relationships and enforcing constraints to preserve the structural and multilayered nature of the networks.

A key strength of L-HetNEtAligner is its ability to align nodes even when there is limited overlap in node attributes or when the topologies of the layers vary significantly. This capability makes it particularly suitable for applications in computational biology and bioinformatics, such as aligning molecular interaction networks, discovering conserved pathways across species, or integrating multimodal data in systems biology. Furthermore, the algorithm is scalable and can handle large, complex networks efficiently, providing a valuable tool for exploring the relationships and conserved patterns in heterogeneous systems.

### 4.3 MuLAN

MuLAN (Multilayer Network Alignment) Milano et al. (2024) is an algorithm specifically tailored for aligning multilayer networks that capture the complexity of real-world systems with multiple types of nodes and edges. MuLAN integrates information from both the intralayer and interlayer connections to establish alignments between nodes across networks. The algorithm employs a novel scoring system that combines topological similarity, based on network structure, with attribute-based similarity, considering node labels or properties.

The alignment process in MuLAN starts by constructing similarity matrices for each layer, where nodes are compared based on their local and global network properties, such as degree and centrality, as well as their connections across layers. These matrices are then iteratively refined using a heuristic optimization strategy that ensures consistency in alignments across layers and maximizes an objective function designed to preserve the structural and functional relationships of the original networks.

Ability of MuLAN to simultaneously handle multiple types of interactions and heterogeneity makes it highly effective for applications in biology, such as aligning multilayer molecular networks to identify conserved pathways, comparing interactomes across species, or integrating multi-omics datasets. The algorithm’s scalability and adaptability also allow it to be applied to other domains, such as social networks or transportation systems, where multilayer structures are prevalent.

MuLAN has significant biological relevance, as it enables the systematic comparison and integration of complex biological systems represented as multilayer networks. In the context of systems biology, many phenomena involve interactions at multiple levels, such as protein-protein interactions, gene regulatory networks, metabolic pathways, and phenotypic traits. MuLAN’s ability to align these multilayer networks provides a powerful framework for uncovering conserved patterns, functional modules, and cross-species relationships that might otherwise remain hidden.

One of the primary applications of MuLAN in biology is the identification of conserved pathways across species. By aligning multilayer molecular networks, MuLAN can detect functional modules or signaling cascades that are preserved evolutionarily, offering insights into fundamental biological processes. These conserved elements can guide the identification of key regulatory mechanisms or essential genes involved in critical cellular functions.

In addition to evolutionary studies, MuLAN has proven valuable in integrating multi-omics data. By aligning networks that incorporate different layers of biological information, such as transcriptomics, proteomics, and metabolomics, MuLAN facilitates the discovery of cross-layer interactions that contribute to complex traits or diseases. This integrative approach can reveal novel gene-disease associations, highlight biomarkers for disease diagnosis, or identify candidate genes for drug targeting.

Furthermore, MuLAN’s capacity to analyze heterogeneous biological networks makes it particularly relevant for precision medicine. For example, aligning patient-specific networks to reference models can uncover individual variations in molecular interactions, aiding in personalized treatment strategies. Similarly, by aligning drug-target interaction networks with disease-specific molecular networks, MuLAN can predict potential drug repurposing opportunities or identify off-target effects.

Overall, MuLAN provides a comprehensive and scalable tool for leveraging multilayer network data to generate biological knowledge, offering a pathway to better understand the interconnected nature of molecular systems, uncover conserved biological principles, and translate these insights into practical applications in medicine and biotechnology.

Table 4 summarizing the main characteristics of L-HetNEtAligner and MuLAN in terms of their features, usage, and capabilities.

**TABLE 4:** Alignment tools features.

Tool	Function	Data input	Output	Key features	Use case
L-HetNEtAligner	Alignment of Heterogeous Networks	Heterogeous Networks	Local Alignment as modules	Focuses on heterogeneous data such as gene, protein, diseases	Discovering groups of related entities should have a similar biological role or share some functions
MuLAN	Alignment of Multilayer Networks	Multilayer Networks	Local Alignment as modules	Focuses on multilayer system such as gene, protein, diseases and drugs	Discovering candidate drug-disease associations and, consequently, of extracting new knowledge from multilayer networks

Main characteristics of L-HetNEtAligner and MuLAN.

## 5 Pathway enrichment analysis for pharmacogenomics

Pathway Enrichment Analysis (PEA) is a computational approach used to identify biological pathways that are significantly associated with a given set of genes or proteins. By leveraging curated pathway databases, such as KEGG (Kanehisa et al., 2017), Reactome (Fabregat et al., 2018), or BioCarta (Nishimura, 2001), PEA helps to uncover functional relationships and molecular mechanisms underlying complex biological functions. This method statistically evaluates whether a predefined group of genes, such as those differentially expressed in a study, is overrepresented in specific pathways compared to random distribution. PEA is widely used in PGx to interpret genetic variants’ impact on drug metabolism, toxicity, and efficacy, ultimately aiding in developing personalized therapeutic strategies.

### 5.1 BiP

BioPAX-Parser (BiP) (Agapito et al., 2020) is a software tool designed to streamline the exploration and enrichment analysis of biological pathways encoded in the BioPAX format. BiP application is available at https://gitlab.com/giuseppeagapito/bip. Unlike other tools, BiP provides a user-friendly graphical interface, eliminating the need for extensive programming skills to parse or analyze pathways. It enables users to easily extract and annotate pathway data, identifying associated genes and proteins. BiP can conduct pathway enrichment analysis (PEA) using data from various BioPAX-compliant databases. It employs statistical methods, including hypergeometric tests, to enrich gene lists. Additionally, BiP incorporates several statistical correction methods to minimize type I and II errors caused by multiple comparisons, such as false discovery rates (FDR) and the Bonferroni correction, ensuring accurate enrichment results. BiP is designed for efficiency, employing advanced data management techniques and multi-threaded processing to ensure fast and scalable analyses. Its intuitive interface makes it accessible to non-programmers, allowing users to visualize, explore, and analyze pathway components effortlessly, all while supporting a wide range of data formats and sources.

### 5.2 PathDIP

PathDIP (Rahmati et al., 2017) is a comprehensive and integrative pathway database designed to facilitate the analysis of biological networks across multiple species, including humans, model organisms, and domesticated animals. By consolidating core pathways from major curated databases and incorporating gene-pathway associations inferred from physical protein interactions, PathDIP enhances the understanding of molecular processes underlying various biological functions and diseases. The platform allows users to input proteins or genes, specify an organism, and select a preferred database for analysis, making it a flexible and user-friendly tool for pathway exploration. It is freely accessible at http://ophid.utoronto.ca/pathDIP. PathDIP generates pathway enrichment results and functional annotations, which are provided in a structured tab-separated format for easy downstream analysis. These results can be visualized as interactive diagrams or tables, facilitating intuitive data interpretation. The platform supports multiple programming environments, including Java, R, and Python, enabling seamless integration into diverse bioinformatics pipelines. By aggregating data from numerous well-established pathway databases, PathDIP offers a robust resource for conducting pathway enrichment analysis, revealing intricate molecular interactions, and elucidating biological mechanisms relevant to pharmacogenomics, precision medicine, and disease research.

### 5.3 GSEA

Gene Set Enrichment Analysis (GSEA) (Subramanian et al., 2007) is a widely used computational method for identifying biologically meaningful patterns in gene expression data. It is designed to analyze datasets with two predefined classes, such as Responder vs Non-Responder or Treated vs Non-Treated, making it particularly useful for differential expression studies. GSEA is best suited for cases where ranks for all genes in a dataset are available, allowing for robust statistical enrichment analysis. The tool is freely accessible at https://www.gsea-msigdb.org/gsea/login.jsp. GSEA features both a user-friendly graphical interface for non-programmers and a command-line interface for bioinformatics experts, ensuring accessibility across different levels of expertise. It supports various input and output formats, enabling seamless integration with diverse bioinformatics workflows. The core analysis methodology relies on enrichment scores to assess the overrepresentation of specific gene sets and employs multiple testing correction methods to enhance statistical reliability. Additionally, GSEA retrieves pathway annotations from several well-established databases. It also allows users to incorporate custom pathway databases in Gene Matrix Transposed (GMT) format, offering flexibility for domain-specific research.

Table 5 summarizes the main characteristics of BiP, PathDip and GSEA in terms of their features, usage, and capabilities.

**TABLE 5:** Pathway enrichment tools features.

Tool	Function	Data input	Output	Key features
BiP	Parses and analyzes BioPAX pathway data	BioPAX (XML format) + gene lists	Pathway components, network structures	Multi-threaded processing, intuitive visualization, pathway enrichment analysis
PathDIP	Integrates and predicts gene-pathway associations	Protein/gene lists	Enrichment results, pathway annotations	Combines curated pathways with computational predictions, supports multiple organisms
GSEA	Identifies enriched gene sets in expression data	Gene expression matrices + class labels	Ranked gene sets, enrichment scores	Uses enrichment scores and multiple correction methods, supports predefined/custom gene sets

Comparison of BiP, PathDIP, and GSEA in terms of their features, usage, and capabilities.

## 6 A comprehensive user guidelines to perform PGx data analysis

Pharmacogenomic (PGx) data analysis involves identifying genetic variations that affect drug metabolism, efficacy, and toxicity. Below is a general step-by-step guide to performing a comprehensive PGx data analysis.

1. Data retrieval regards the collection of raw genomic data obtained from various genotyping platforms like DMET chips, SNP microarrays and whole-genome sequencing. Following data collection, rigorous quality control (QC) measures must be applied. This involves checking the dataset for missing genotype calls, which can affect the reliability of downstream analyses. Low-quality samples or genetic markers with low call rates should be filtered out to maintain the integrity of the dataset. The final step in data preparation is the standardization of input formats. Raw data must be converted into universally recognized formats such as Variant Call Format (VCF), textual tables of SNPs comma-separated value (CSV), or formats compatible with tools like PLINK. This standardization ensures interoperability between various analysis tools and facilitates seamless downstream analyses, enabling accurate and reproducible results.
2. PGx-Related SNPs identification. The analysis begins with exploratory data analysis, i.e., data preprocessing, which provides an overview of the dataset’s structure, distribution of variables, and potential anomalies. This phase often includes visualizing genotype frequencies, identifying and handling missing data, filter low quality samples, detection and removing of duplicated samples, checking and imputation of missing genotype calls. After data preprocessing, statistical, machine learning or both analysis can be conducted to identify significant associations between genetic variants and drug response.
  a. Statistical tests can be conducted using manual methodology like Fisher’s Test, chi-squared test or using software framework like DMET-Analyzer, cloud4SNP or OSAnalyzer to automatically figure out significant statistical association between genetic variants and the phenomena under investigation.
  b. ML methods enhance the ability to detect complex patterns and interactions within PGx datasets. Supervised learning models such as support vector machines (SVMs), random forests, neural networks can be trained to predict phenotypic outcomes or classify individuals based on their genetic profiles. For unsupervised learning tasks, clustering, association rules implemented in DMET-Miner may be applied to identify multiple genetic or phenotypic characteristics.

Obtaining as a result a list of genotypic data genes, SNPs affecting the phenotype and the drug response or the adverse responses.

3. Network Analysis. The previously identified genes or SNPs can serve as the foundation for conducting a network analysis, a robust approach for uncovering biological interactions. This involves mapping these genes onto molecular interaction databases such as STRING or Reactome to explore their roles within the broader biological network. To enhance the relevance of the network, the immediate neighbors of these DMET genes are identified through network alignment. This process expands the initial set of genes to include first-order interactions, which are essential for contextualizing their biological functions. Network alignment is a critical step that provides insights into how these genes operate within interconnected pathways. Network alignment ca be performed in two different ways:
  a. Global network alignment aims to find a one-to-one mapping between all nodes of two networks. The primary goal is to maximize the overall similarity of the aligned networks, focusing on preserving structural and functional relationships across the entire network.
  b. Local network alignment focuses on identifying smaller, highly similar substructures between networks. Unlike global alignment, it does not aim to map all nodes but rather seeks to find regions of high similarity.
4. Pathway enrichment analysis (PEA) identifies biological pathways that are significantly overrepresented within the network of neighboring genes. This network is refined by applying filters to eliminate redundant genes and interactions with low confidence or limited biological relevance, thereby ensuring that only meaningful associations are retained. Tools such as BiP, PathDIP, StringAPP, and Reactome can be employed to perform PEA effectively. The analysis outputs a list of key biological pathways directly linked to drug metabolism and transport processes, highlighting pathways that show statistically significant enrichment beyond what would be expected by chance. This approach facilitates the identification of critical hubs and novel pathways that may influence drug response, contributing to a deeper understanding of the PGx landscape.

**FIGURE 2:**
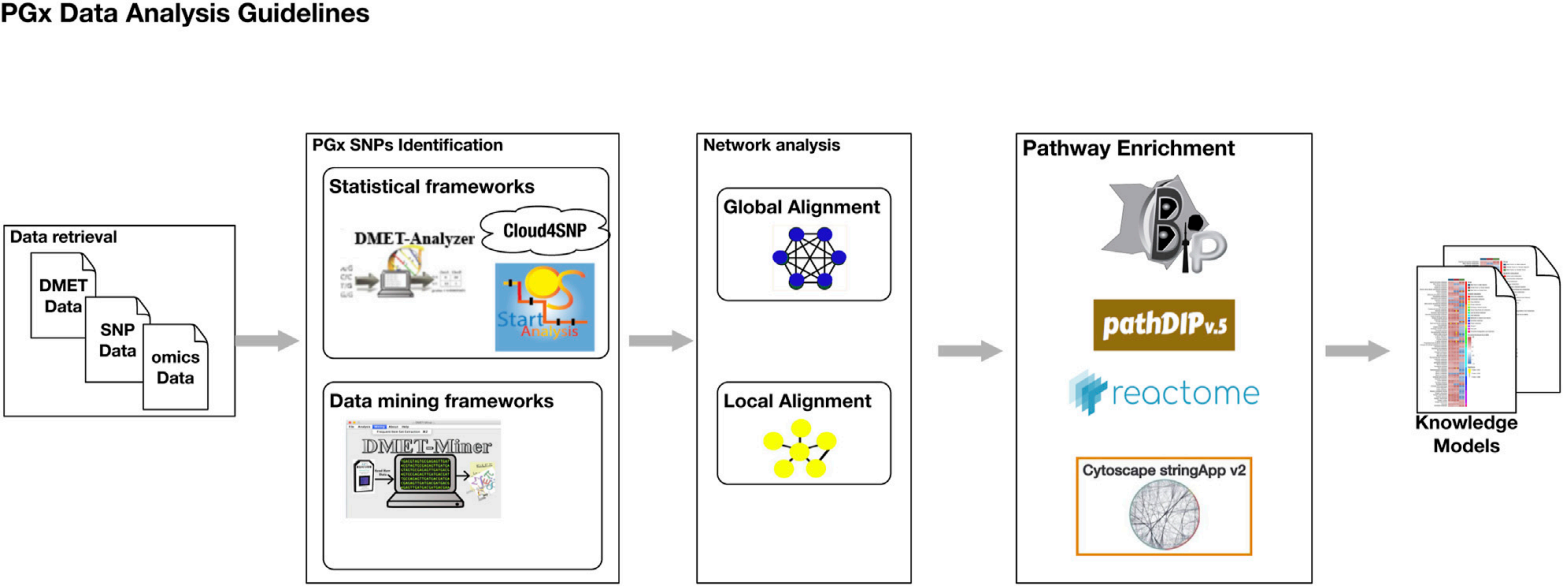
The graphical representation of the PGx data analysis guidelines.

Figure 2 illustrates the key steps of the PGx data analysis guidelines through a graphical representation.

Finally, the results are interpreted in the context of existing biological knowledge and clinical guidelines. This involves mapping significant genetic variants to PGx databases such as PharmGKB or ClinVar and integrating findings with pathway analyses to provide a mechanistic understanding of drug-gene interactions. These steps collectively form a comprehensive framework for leveraging statistical and machine learning approaches in pharmacogenomics, enabling the translation of genetic insights into personalized therapeutic strategies.

### 6.1 Integrative analysis of genomic data using bioinformatic platforms: a real use case of the methodology

**FIGURE 3:**
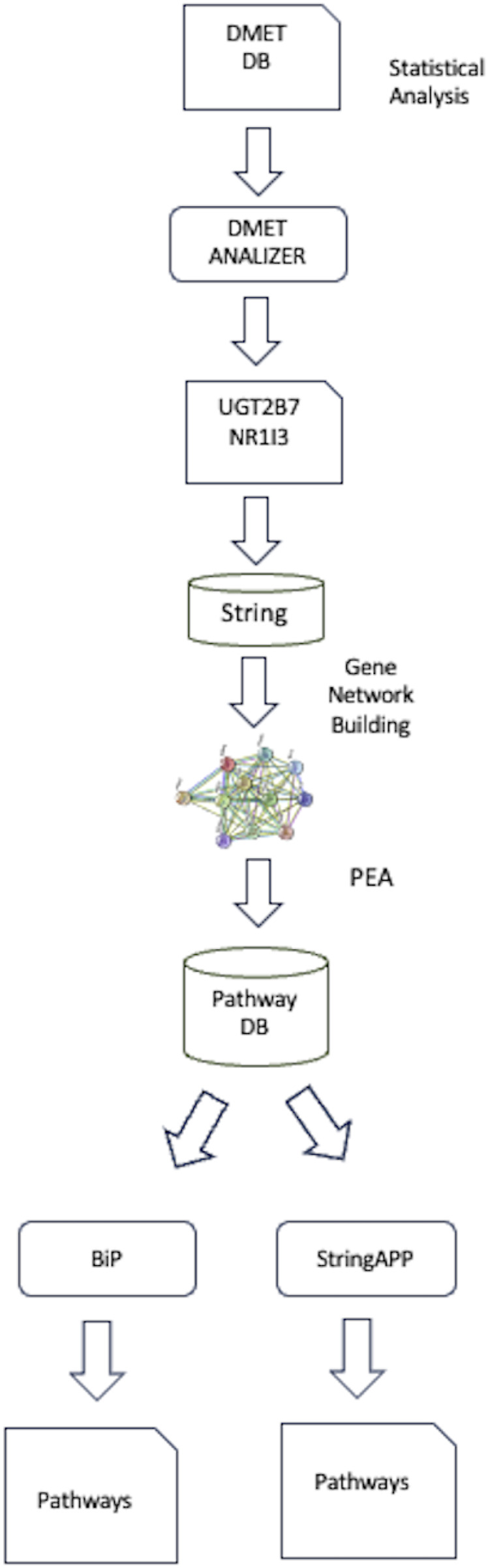
Workflow of analysis.

In this section we present an example of integrated analysis of identified SNPs correlated to taxane-related peripheral neurotoxicity (TrPN) in a retrospective case-control study on breast cancer (BC) patients (Arbitrio et al., 2019) based on the guidelines above described. Using DMET genotyping, we feed DMET-Analyzer with the raw DMET SNPs data set, to identify specific SNPs within these genes associated with TrPN, figuring out these tow genes UGT2B7 and NR1I3. The NR1I3 and UGT2B7 genes have been implicated in taxane neuroprotection due to a defect in the drug’s glucuronidation/clearance process related to ultrametabolizer phenotype with probable loss of efficacy, suggesting new potential biomarkers correlated to taxane neurotoxicity. These potential biomarkers could allow stratification of BC patients with interindividual TrPN predisposition for tailored prescription (Arbitrio et al., 2019). To further explore their biological significance, a network-based analysis was conducted using the STRING database to construct a gene interaction network, focusing on genes functionally related to UGT2B7 and NR1I3. Following, PEA was performed to link these genes to biological pathways involved in drug metabolism. The identified pathways were then modeled using a multilayer network approach, which allowed the integration of multiple interconnected pathways to better understand the complex roles of these genes in disease mechanisms and drug responses. This methodology highlights UGT2B7 and NR1I3 as potential biomarkers for predicting taxane toxicity, estrogen processing and neuroprotection and optimizing treatment strategies in precision medicine. Figure 3 summarizes the main step fo analysis, for complete details on methodology see Agapito et al. (2024).

## 7 Future perspectives

Bioinformatics in PGx studies present several promising advancements, particularly in omics data analysis. One of the most significant developments will be the integration of multi-omics data, encompassing genomics, transcriptomics, proteomics, and metabolomics (Wang and Agapito, 2025). This holistic approach will allow researchers to unravel complex molecular interactions influencing drug metabolism, efficacy, and adverse reactions. By merging multi-omics datasets, PGx will transition toward a more precise and individualized understanding of drug responses, ultimately enhancing personalized medicine (Guzzi et al., 2016).

Clinical trial results are essential for advancing PGx, providing insights that help regulatory agencies and clinicians optimize therapies. However, strict enrollment criteria can limit the generalizability of findings and may not fully reflect the complexity of drug responses in real-world settings (You et al., 2025). Factors such as genetic variability, comorbidities, polypharmacy, and environmental influences are often underestimated.

Integrating PGx data with real-world evidence can greatly enhance biomarker discovery and the development of personalized medicine through the application of AI. Thus, as AI and ML technologies continue to advance, its role in PGx may help bridge the gap between controlled clinical trials and the complexities of real-life medical practice. This could lead to more precise and inclusive therapeutic strategies. Advanced ML algorithms, including deep learning frameworks, will facilitate the identification of complex genetic interactions and improve predictions of patient-specific treatment outcomes. These computational models will be instrumental in refining PGx biomarkers, optimizing drug selection, and minimizing adverse drug reactions, enhancing therapeutic effectiveness (Scionti et al., 2022).

In addition to AI-driven approaches, network-based analysis and systems biology methodologies will be crucial in advancing PGx studies. Network analysis, pathway enrichment techniques, and multilayer network models will enable researchers to decipher intricate drug-gene interactions and uncover novel therapeutic targets. By integrating these methodologies, it will be possible to develop a more exhaustive awareness of the molecular mechanisms underlying drug responses.

Another key area of progress will be the development of user-friendly bioinformatics platforms designed to facilitate genomic data analysis for both researchers and clinicians. These platforms will integrate curated PGx databases, statistical frameworks, and visualization tools, allowing non-specialists to interpret complex genetic data more efficiently. Such advancements will promote the widespread adoption of PGx in clinical settings and enhance its accessibility for healthcare professionals.

Cloud computing and big data analytics are transforming the field by offering scalable solutions for storing, processing, and sharing large-scale PGx datasets (Crespo-Cepeda et al., 2019). Cloud-based bioinformatics tools will enable researchers across different institutions to collaborate more efficiently, ensuring the rapid dissemination of PGx findings and fostering global initiatives in precision medicine.

The future of PGx will also be shaped by its integration into regulatory settings and clinical decision-making processes. As PGx research continues to evolve, regulatory agencies such as the FDA and EMA are expected to incorporate bioinformatics-driven PGx tools into drug labeling recommendations. This regulatory support will facilitate the transition of PGx from research to routine clinical practice, ultimately improving drugs safety and efficacy.

While advancements have been made, addressing ethical, legal, and social challenges for the responsible implementation of PGx technologies is crucial. Issues related to genomic data privacy introduce new challenges to face, like in Cloud Computing (Agapito and Cannataro, 2023), equitable access to PGx testing, and potential biases in AI-driven analyses must be carefully managed to ensure that PGx advancements benefit diverse patient populations without reinforcing healthcare disparities.

Bioinformatics in PGx is expected to grow significantly due to advancements in AI, big data, network analysis, and cloud computing. These advancements will enhance our understanding of genetic contributions to drug responses and pave the way for more effective, individualized therapeutic strategies in clinical practice.

## 8 Conclusion

This study underscores the critical role of PGx data analysis in advancing precision medicine, highlighting how integrative analysis through bioinformatics tools and methodologies could allow in unraveling the genetic underpinnings of drug response variability. By integrating statistical, machine learning, network analysis, and pathway enrichment approaches, we demonstrated how complex datasets can be effectively managed and analyzed to derive actionable insights.

The investigation of UGT2B7 and NR1I3 genes convey the potential of such approaches to identify predictive biomarkers for drug toxicity and efficacy. Specifically, the integration of bioinformatic frameworks allowed the identification of these genes as critical players in taxane-related peripheral neurotoxicity and estrogen metabolism, paving the way for personalized treatment strategies in breast cancer management. The results emphasize the necessity of continuous innovation in bioinformatics tools to address the challenges posed by high-dimensional, heterogeneous PGx datasets.

Also the recently development of user-friendly PGx platforms for data analysis might play a crucial role in advancing precision medicine making it easier to understand and interpret PGx data. The simplification of complex information starting from the integration of curated databases and clinical guidelines and providing actionable insights for clinicians and researchers could pave the way for a future healthcare tailored to each individual’s unique genetic makeup (Duong Nguyen et al., 2025; Borbón et al., 2025; Yuan et al., 2025, Yuan et al., 2024). This work not only contributes to the understanding of PGx variability but also provides a comprehensive framework for translating genetic insights into clinical practice, offering promising opportunities for improving therapeutic outcomes through tailored interventions. Additional prospective research could further confirm the strategic and therapeutic value of integration of PGx bioinformatic strategies to improve patient outcomes, reduce healthcare costs, and tailored drug prescription.

## Data Availability

The original contributions presented in the study are included in the article/supplementary material, further inquiries can be directed to the corresponding author.
